# GPs’ and practice nurses’ views on their management of paediatric anxiety problems: an interview study

**DOI:** 10.1186/s12875-022-01802-y

**Published:** 2022-09-12

**Authors:** Lukas B. M. Koet, Jessie J. M. Bennenbroek, Annouk Y. S. Bruggeman, Evelien I. T. de Schepper, Arthur M. Bohnen, Patrick J. E. Bindels, Heike Gerger

**Affiliations:** 1 Department of General Practice, ErasmusMC University Medical Center, PO Box 2040, 3000 CA Rotterdam, the Netherlands; 2 Care and Public Health Research Institute, Maastricht University, Maastricht, the Netherlands

**Keywords:** Children, Adolescents, Anxiety problems, General practice, Practice nurse, Management, Treatment

## Abstract

**Background:**

Anxiety problems are common in both children and adolescents, and many affected children do not receive appropriate treatment. Understaffing of mental healthcare services and long waiting lists form major barriers. In the Netherlands, practice nurses have been introduced into general practice to support general practitioners (GPs) in the management of psychosocial problems. In this study we investigated the views of GPs and practice nurses on their management of paediatric anxiety problems.

**Methods:**

We performed an exploratory study using semi-structured interviews with 13 GPs and 13 practice nurses in the greater Rotterdam area in 2021. Interviews were transcribed and coded into topics, which were categorized per research question.

**Results:**

In their management of paediatric anxiety problems, both GPs and practice nurses try to explore the case and the needs of affected children and their parents. GPs rarely follow up affected children themselves. They often refer the child, preferably to their practice nurse. Practice nurses regularly initiate follow-up consultations with affected children themselves. Practice nurses reported using a variety of therapeutic techniques, including elements of cognitive behavioural therapy. In more severe cases, practice nurses refer the child to external mental healthcare services. GPs reported being satisfied with their collaboration with practice nurses. Both GPs and practice nurses experience significant barriers in the management of paediatric anxiety problems. Most importantly, long waiting lists for external mental health care were reported to be a major difficulty. Improving cooperation with external mental healthcare providers was reported to be an important facilitator.

**Conclusions:**

In their management of paediatric anxiety problems, GPs and practice nurses experience major challenges in the cooperation with external mental healthcare providers and in the long waiting lists for these services. GPs and practice nurses believe that thanks to their shared approach more children with anxiety problems can remain treated in general practice. Future research is needed to evaluate the treatment outcomes of the shared efforts of GPs and practice nurses in their management of paediatric anxiety problems.

**Supplementary Information:**

The online version contains supplementary material available at 10.1186/s12875-022-01802-y.

## Introduction

Anxiety disorders are among the most common mental health problems in children and adolescents [1, 2]. Anxiety disorders are a significant burden for affected children and their next of kin [3, 4]. Furthermore, paediatric anxiety disorders are associated with psychosocial problems in adulthood [5–7]. Fortunately, effective therapies exist [8, 9], e.g. cognitive behavioural therapy (CBT), which has been shown to improve long-term outcomes in affected minors [10–12]. Although paediatric anxiety disorders occur frequently, affected minors often do not receive adequate therapy [13–15]. Previous research identified three main barriers to appropriate care. Firstly, affected minors and their caregivers often do not seek help [16–19]. Secondly, key people in the recognition of anxiety disorders, e.g. teachers and general practitioners (GPs), often do not recognize signs of underlying anxiety [16, 20–22]. Thirdly, in many countries mental health care is under major pressure, with understaffing and long waiting lists forming another barrier to obtaining appropriate help [23–25].

To tackle this last barrier, initiatives have been taken in many countries to improve access to youth mental health care [26–30]. Dutch policy-makers have made significant changes to the organization of youth mental health care in the past years to make it more accessible and cost-effective. In the Netherlands, mental health care for children (≤17 years) is free of charge. Youth mental health care was recently decentralized and it now falls under the responsibility of local municipalities (Dutch Youth Act 2015). As part of this process, local ‘neighbourhood teams’ have been established to provide or organize care for children and their caregivers. At the same time, policy-makers have sought to integrate mental health services into general practice.

Dutch GPs have a gatekeeper role and provide most primary care for children. GP care is complemented by the work of youth physicians, who work in schools or institutions and mostly provide preventive care, such as vaccinations and screening. In addition, paediatricians provide specialized medical care for which a referral from a GP is needed. To access mental health care, families can also approach a local ‘neighbourhood team’ or youth-care institution (*Centrum Jeugd en Gezin*). Since 2008 GPs can employ ‘mental health practice nurses’ (MHPNs) to assist them in management of psychosocial problems [31]. Although MHPNs are primarily involved in care for adults, some MHPNs also provide services to children and adolescents. In 2015, a specialized position of ‘youth mental health practice nurse’ (YMHPN) was introduced into general practice. These YMHPNs are professionals with a background in youth care who provide help by examining, screening, giving short-term treatment and referring minors with psychosocial problems [32].

Concerning the treatment of paediatric anxiety problems and disorders in primary care settings, a few small-scale studies have been performed internationally, showing promising results [33–35]. In the Netherlands, GPs reported being satisfied with the presence of YMHPNs in their practice [32]. To our knowledge, however, no studies so far have evaluated the current management of paediatric anxiety problems either by GPs alone, or by GPs in cooperation with practice nurses. Therefore, in this study we evaluated the experiences of GPs, MHPNs and YMHPNs with their combined management of children (≤17 years) with anxiety problems. We addressed the following research questions. How do GPs, MHPNs and YMHPNs currently manage paediatric anxiety problems? What are barriers and facilitators in this process? What changes in the management of paediatric anxiety problems have taken place since the involvement of MHPNs and YMHPNs? This study focused on anxiety problems on symptomatic description, rather than focusing on individual anxiety diagnoses. This is in line with GP practice, because GPs often prefer describing patient symptoms rather than giving formal diagnoses in the context of paediatric anxiety.

## Methods

### Participants and study design

Based on the literature and previous related studies, we aimed to include 10–13 GPs and 10–13 YMHPNs for semi-structured interviews [36–40]. We included 13 GPs and 13 MHPNs or YMHPNs from 15 practices in our study. Two junior researchers conducted the interviews in July and August 2021 (GPs by JB, MHPNs by AYSB) after following internal training on interviewing and qualitative analysis.

We intended to reach a sample of GPs and YMHPNs involved in managing paediatric psychosocial problems from one geographical region only (to control for regional differences in healthcare organization). Because the position of YMHPN was only recently introduced, the exact job requirements and financing of the position are not well-established. Although YMHPNs work in general practices, they can be employed either by GPs or by the local municipality. YMHPNs are expected to have relevant work experience e.g. as a MHPN, psychiatric nurse, social worker or psychologist. However, the GPs or the local municipality themselves decide which qualifications are exactly required when hiring a MHPN. Additionally, it became clear that in many practices MHPNs were involved in the management of both adults and minors. Therefore, we decided to include both YMHPNs only managing minors, and MHPNs managing both adults and minors, which best reflects the current practice. We will refer to our study group as MHPNs.

We invited 30 general practices from the greater Rotterdam area to take part. First, we sent an e-mail with information on the study and its goals, and subsequently we contacted them by phone to confirm participation. Fourteen of the 30 practices were contacted via our academic GP network (PRIMEUR), and the others were identified via their practice websites, or via the professional networks of the researchers.

The greater Rotterdam area has a population with diverse backgrounds (e.g. in cultural and socio-economic characteristics) and includes urban and more rural regions. Sampling was purposeful in the sense that only practices with MHPNs involved in managing minors were invited. We did not define additional inclusion criteria with respect to other characteristics of the practice, GPs or MHPNs, in order to include a wide range of practices with varied patient populations reflecting the population of the greater Rotterdam area. Inadvertently, one GP was included in the sample even though he did not employ a MHPN who managed minors. Relevant information from this interview was included in our analyses for the research questions not specifically related to the collaboration between the GP and MHPN.

Reasons for not participating were: no response or final decision (eight practices), no time (three practices), currently no MHPN managing minors (three practices), no reason (one practice). One interviewer knew one GP before the interview because of a previous internship in their practice. No other private or work relationships existed between the interviewers and interviewees before study commencement.

### Materials

#### Survey

We gathered relevant characteristics of the interview partners using a short online survey (LimeSurvey, version 2.06) in order to reduce the time required for the actual interview. This pre-interview survey contained questions about practice characteristics, participants’ characteristics, and their experience with diagnosing and treating paediatric anxiety problems (Supplementary file S4).

#### Interview

Semi-structured interviews with GPs and MHPNs provided the main data for our analyses for answering our research questions. The interviews addressed the GP’s and MHPN’s management of children presenting with anxiety problems, the barriers and facilitators they experienced, and the changes in management since the involvement of MHPNs in managing minors with psychosocial problem. As the study was conducted in July to August 2021, possible influences of the ongoing Covid-19 pandemic on the study were checked with two interview questions. An interview guide was constructed, consisting of open interview questions (Supplementary file S5). The interviews were pilot-tested with two GP researchers, which led to minor changes in the phrasing and order of the interview questions.

#### Vignette

Each interview started with the interviewee silently reading a vignette describing a child and their mother consulting their GP with symptoms as manifestations of underlying anxiety problems (Supplementary file S5). This served as a reference case onto which the interviewees could project their responses during the interview. To develop the vignette, two clinical cases were formulated as potential vignettes (AYSB/JB/HG) based on a literature review. Experts were then consulted (three GPs and two psychologists/psychotherapists), and one of the two cases was chosen based on the experts’ feedback.

### Data collection

After they had agreed to participate in the study, participants were invited to complete the online survey and interview dates were planned. Depending on the participant’s preference, the interviews were performed at the participants’ practice or via encrypted video-calls, and in two cases by phone due to technical problems. Interviews were performed one-on-one, with the exception of one interview, which was conducted with two MHPNs at their explicit request. Interviews took approximately 30 minutes (GPs: 20–30 minutes, MHPNs: 20–45 minutes), were audio-recorded and transcribed verbatim. Participants’ names were pseudonymized and identifying words were removed. Transcripts were not returned to participants.

### Analysis

We used descriptive statistics (SPSS version 25.0) for the analysis of the characteristics of the interview partners which we gathered using an online survey (see Table 1).

**Table 1:** Summary of characteristics of the interview participants

	13 GPs	13 MHPNs
Sex	8 male (61.5%), 5 female (38.5%)	1 male (7.7%), 12 females (92.3%)
Mean Age (SD)	46.6 (6.5) years	47.1 (10.1) years
Work experience in years (SD)	GP since 16.7 (6.7) years	MHPN since 3.7 (2.9) years
Full-time (≥36 hours)	7 Full-time (53.8%), 6 Part-time (46.2%)	13 Part-time MHPN (100%)
General Practice Social economic status	Social economic status: 76.9% normal, 14.4% low, 7.7% high	Social economic status: 84.6% normal, 14.4% low
MHPN patient population	N.a.	Manages exclusively children/adolescents: 30.7% Manages children, adolescents and adults: 69.3%
Experience diagnosing anxiety problems	Much: 14.4% Neutral: 46.2% Limited: 30.8% Very limited: 7.7%	Much: 7.7% Neutral: 38.5% Limited: 46.2% Very limited 7.7%
Experience treating anxiety problems	Neutral: 61.5% Limited: 14.4% Very limited: 23.1%	Very much: 7.7% Much: 30.7% Neutral: 23.1% Limited: 23.1% Very limited: 15.4%
Possibility to refer to ‘neighbourhood team’	9 GPs (69.2%)	11 MHPNs (84.6%)
Usual approach to child with anxiety problems	Investigate the problem yourself after which referral for treatment: 53.8% Direct referral for additional examination and treatment: 23.1% Wait-and-see: 7.7% Other: 14.4%	Investigate the problem myself and start treatment: 23.1% Investigate the problem yourself after which referral for treatment: 46.2% Direct referral for additional examination and treatment: 7.7% Other: 23.1%
Referral to (most commonly)	MHPN: 53.8% Child psychologist 30.8% Specialized mental health: 15.4%	Child psychologist: 53.8% Specialized mental health: 23.1% Neighbourhood team: 7.7% Other: 15.4%

Transcripts of the interviews were coded using an online coding tool (QCAMAP.org, v.1.0.9) [41]. We read the interview transcripts sentence by sentence and assigned a code (topic) to each unit of information relating to one of our research questions in line with the coding procedure described by Boeije [42]. The coding was data-driven and new codes were added to the coding tree if a new topic was mentioned in an interview. Subsequently, we categorized the emerged topics into a hierarchy of main topics, topics, and subtopics per research question (See supplementary file S2). The initial coding of the interview transcripts was performed by one junior researcher (GPs by JB, MHPNs by AYSB). During the coding process, the emerging code trees were regularly reviewed and discussed in group consensus meetings (AYSB/JB/HG/LK). Finally, all interviews were re-read and all codes were checked (LK), which led to minor changes in the labelling of individual topics. The final code tree with main topics, topics and subtopics was checked and approved by the research team (AYSB/JB/HG/LK).

Our main research goal was to explore first, the current management of paediatric anxiety problems by GPs and MHPNs, as well as second, barriers and facilitators experienced by GPs and MHPNs. Therefore, we chose to analyse the qualitative data close to the original data (i.e., the interview transcripts) by summarising interview statements into relevant topics and by organizing the emerged topics into a hierarchy of main topics, topics and subtopics (See supplementary file S2). More in-depth analyses using transformation and interpretation of data regarding latent meaning and content was not the goal of our research.

Data saturation occurred in the interviews with GPs between interviews 9–10, and in the interviews with the MHPNs between interviews 11–12. Nevertheless, all recruited participants were interviewed in order to check for the robustness of data saturation.

### Data protection and ethics

Ethical approval was obtained from the ErasmusMC’s ethics board (MEC-2021-0406) before study commencement. Participants were informed about the confidentiality and data security agreements. Participants gave their consent for participation in the online survey and at the beginning of each interview. Participation in the study was voluntary and there was no financial compensation. Data was stored securely at the Department of General Practice, ErasmusMC. In this study, we adhered to the COREQ guidelines (Supplementary files S6).

## Results

### Participants

In total 13 GPs (8 men, 5 women) participated in our study. They had a mean age of 46.6 (SD 6.5) and 16.7 years of work experience as a GP (SD 6.7 years). In total, 13 MHPNs (1 man, 12 women) participated with a mean age of 47.1 (SD 10.1), and with a mean of 3.7 years of work experience as MHPN (SD 2.9 years). Four MHPNs (30.8%) worked exclusively with children and adolescents. Nine MHPNs (69.2%) managed both adults and minors. Table 1 summarizes the survey findings.

### Themes

The interviews conducted with GPs revealed 655 text elements, ordered in 90 topics. Eleven main topics, 65 topics, and 4 subtopics referred directly to our research questions. The 10 remaining topics provided additional information (e.g. referring to the control questions due to the Covid-19 pandemic). In the MHPN interviews we coded 625 text elements, which led to the identification of 105 topics. Ten main topics, 68 topics, and 14 subtopics referred directly to the three main research questions of our study. The 13 remaining topics provided additional information (Supplementary files S2).

#### How do GPs and MHPNs currently manage paediatric anxiety problems?

In the shared management of paediatric anxiety problems, MHPNs take on much of the management, often in an early stage. This is illustrated by a GP and a MHPN explaining their approach to the vignette.

*“But with such a girl, I would be inclined to ask the YMHPN to see the child… to explore for underlying problems.” (GP-5)* “*First, I would explore the case … and possibly I would contact her school … Depending on the severity of the case, I would start (therapeutic) sessions with the child, perhaps together with her mother..” (MHPN-2)*

##### Management of paediatric anxiety problems

In their management of paediatric anxiety problems, GPs and MHPNs try to earn the trust of the child, and explore the severity, duration and background of the problem, and the needs of the child and the parents. GPs and MHPNs investigate the extent to which the anxiety problem influences daily functioning (e.g. at home and in school). Both professional groups pay special attention to the family situation and traumatic events. GPs often investigate associated physical complaints, and eating and sleeping problems. In severe cases, GPs explore the presence of compulsive or suicidal thoughts, self-harm and substance abuse. Both GPs and MHPNs regularly give advice to child and parents to contact schools. GPs rarely contact the school themselves, while MHPNs contact teachers or school social workers more regularly to receive information and to coordinate the management approach. If the anxiety problem is assessed as moderate to severe, most GPs opt for referral of the child. MHPNs refer more severe cases to external mental health services. In mild to moderate cases, MHPNs usually initiate treatment themselves. Helpful factors in the medical decision-making process are shown in Table 2.

**Table 2:** GPs’ and MHPNs’ experienced helpful factors in medical-decision making

GPs’ helpful factors	MHPNs’ helpful factors
Overview of the local social and mental health care Extensive information on case and its context Expertise with anxiety problems^b^ To take sufficient time for exploration^b^ Clear reason of consultation^a^ To have diagnostic certainty^a^ Agreement between GP and parents on management^a^	Knowledge and experience with anxiety problems To take sufficient time for exploration To have a connection with the patient To have a helpful working experience/ professional background Intuition of the MHPN To have possibility to discuss cases with colleagues/GP/other caregivers Overview of the local social and mental health care^a^ To use a therapeutic model^a^

Topics mentioned by GPs and MHPNs

^a^Mentioned by one interviewee

^b^Mentioned by two interviewees

##### Type of treatment

GPs rarely treat paediatric anxiety problems themselves. Some GPs mentioned holding supportive conversations in exceptional cases. Some MHPNs remarked that they have no formal registration for psychological treatment, and prefer defining their treatment efforts as supportive follow-up. Nevertheless, MHPNs reported using several specific therapeutic tools. Firstly, they use psycho-education to explain the physiological function of fear to the children and their parents. MHPNs explicitly mentioned an aim to involve the child’s social network (especially the parents and school), and to encourage children to expand their social network and to continue/start pleasant habits. They help parents to respond to the child’s behaviour in a supportive but not overprotective way. MHPNs mentioned using elements of CBT, explaining (e.g. by using schematic models) the relationship between thoughts, feelings, physical sensations and behaviour, and stimulating children to interpret situations in a neutral or positive way by establishing positive and helpful thoughts. MHPNs also reported using tools such as an evidence-based workbook (Dutch Youth Institute) or anxiety hierarchy models, and encouraging children to set goals using appropriate exercises. Some MHPNs use e-health e.g. online platforms with information, exercises and the possibility for peer support. Several MHPNs also mentioned teaching affected children breathing and relaxation techniques. In certain cases, children are encouraged to seek help from other care professionals, e.g. creative therapists or psychosomatic physiotherapists. Some MHPNs described their treatment as “eclectic”, using methods from different psychological and therapeutic fields.

##### Referral

GPs prefer referring affected children to their MHPN because of the short waiting time and because they can remain involved in such cases. However, GPs and MHPNs believe severe and complex cases are often better treated by external mental healthcare providers. GPs and MHPNs reported several factors which make them more likely to refer affected children (Table 3).

**Table 3:** GPs’ and MHPNs’ reasons to choose for external referral

Reasons for external referral	
Severe anxiety problem	
Suicidality	
Self-harm	
No improvement despite treatment	
(Comorbid) psychiatric disorders	
Complex family situation	
Traumatic experiences	
Indication for extensive diagnostic assessment	
Explicit request for referral	
Child maltreatment^a^	
No availability of / with MHPN^a^	
Indication other type of treatment^b^	
Long duration of anxiety problem^b^	
Young children^b,c^	

Topics mentioned by GPs and MHPNs

^a^Only mentioned by GPs

^b^Only mentioned by MHPNs

^c^Mentioned by two interviewees

##### Cooperation with external mental health services

Communication with other mental healthcare providers was described by GPs as limited, and experienced as difficult. MHPNs reported being much more involved in working with mental healthcare providers. They try to have a good overview and good contacts with the available mental health institutions and social care providers. Most MHPNs said they were in regular contact with the neighbourhood team, and used these contact moments to discuss cases. They refer children to the neighbourhood team if it can offer more appropriate care. More rarely, MHPNs discuss cases with youth care services or child protection services.

**Fig. 1:**
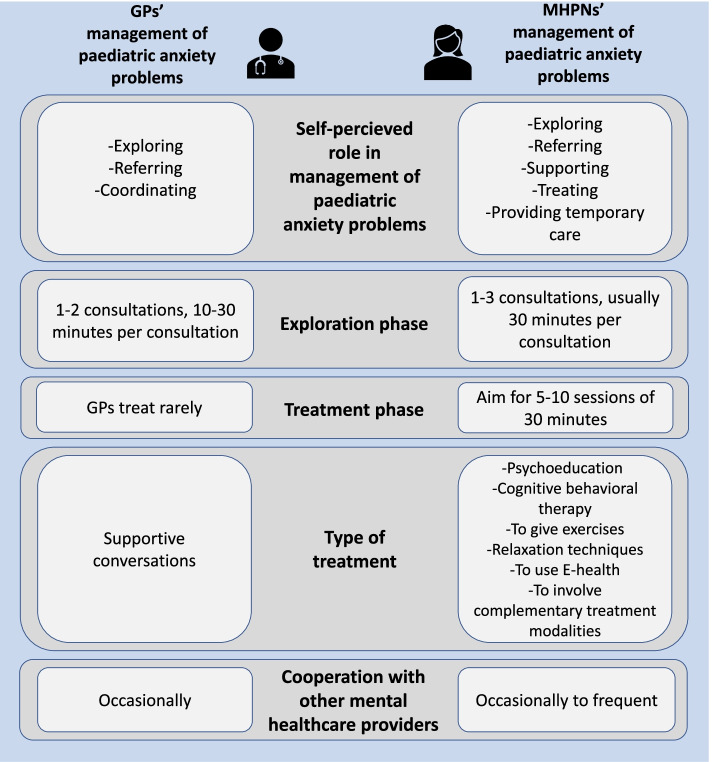
Infographic of the management of paediatric anxiety problems by GPs and MHPNs

Figure 1 summarizes the current management of paediatric anxiety problems by GPs and MHPNs.

#### What are barriers in the management of paediatric anxiety problems?

One the most important barriers experienced by GPs and MHPNs is time constraint, which is illustrated the by the dilemmas mentioned by two interviewees.

*“We notice that (managing children with these kind of problems), takes much more time than just the duration of consultation … We simply do not have that time.” (GP-13) “(after I refer a child) everyone wants me to follow up on the child to ‘bridge’ the waiting time. But if you do this, your agenda will fill up quickly, which makes it difficult to see new cases, so I find that a difficult dilemma.” (MHPN-2)*

##### Barriers experienced by GPs

Overall, GPs believed they lack expertise for managing paediatric anxiety problems. They attributed this to limited training and the low frequency of paediatric anxiety problems in their practice. They also reported that they lacked the time for managing paediatric anxiety problems. Some GPs said that it was a challenge to deal with the family of affected children without harming the physician-family relationship. GPs need to take several complicating factors into account: the different needs of child and caregivers, divorce, parenting styles, and the high expectations of parents. Concerning cooperation with mental health services, GPs often do not know where to refer the child and expect their referrals to be frequently rejected. All GPs are very concerned about the long waiting lists for mental health care, especially for specialized services. Because of limited communication with mental healthcare providers, GPs frequently lose track of the treatment process. Table 4 shows the barriers experienced by GPs and MHPNs.

**Table 4:** Barriers in the management of anxiety problems

GPs’ Barriers	MHPNs’ Barriers
**GPs’ personal and practice barriers**	**MHPNs’ personal and practice barriers**
- Insufficient time - Insufficient expertise/knowledge - No overview/knowledge of mental health care institutions - Concern to damage Patient-Family-Physician relationship^b^ - Limited availably of MHPN/ No other expert in general practice^b^	- Insufficient time in agenda to plan patients - Difficulty choosing best approach/management - Not appropriate care while on waiting list - No experience managing young children with anxiety^b^ - Working with confidential information^a^
**Child and family barriers**	**Child and family barriers**
- Difficult cooperation with child/family - Unrealistic expectations of family^b^ - Disagreement between GP and parents on management^a^ - Child’s barrier to seek help with GP^a^	- Low motivation of children - Complex / non-supportive family situation - Children difficulty expressing themselves / verbal approach less suitable - Unrealistic expectations / non-proactive caregivers^a^ - Difference in needs of child and parents^a^ - Parents with different cultural background^a^
**External mental health care barriers**	**External mental health care barriers**
- Long waiting lists for external mental health care - Obscurity towards efficient referral process - Low quality mental health care^a^	- Long waiting lists for external mental health care - Not covered treatment options by insurance

Topics mentioned by GPs and MHPNs. Main topics in bold

^a^Mentioned by one interviewee

^b^Mentioned by two interviewees

##### Barriers experienced by MHPNs

Some MHPNs reported having insufficient time in their schedules to plan consultations. At times MHPNs experience difficulty deciding on the appropriate management. Some MHPNs reported more difficulty managing young children, for which they lack tools. Differences between the needs of children and their caregivers can also form barriers in their view. Usually caregivers initiate the search for help, but the children are not always motivated for treatment. Furthermore, caregivers can sometimes behave demandingly towards the MHPN without reflecting on their own impact as parents. Complex family situations e.g. divorce and addictions, are additional barriers. The long waiting lists form a major barrier to the access of external mental health care according to MHPNs, which puts them in a dilemma: either they place children on a waiting list without any treatment at all, or they ‘bridge’ the waiting time by supporting these children as well as they can at the expense of seeing other children. Some MHPNs remarked that care by other professionals such as creative therapists or psychosomatic physiotherapists can be useful, but that it is often not reimbursed by health insurance.

#### What are facilitators in the management of paediatric anxiety problems?

Both GPs and MHPNs believed that wide availability of a specialized YMHPNs in general practice facilitates the management of paediatric anxiety such as illustrated in the following statements:

*“… to have an YMHPN in your practice who is available and easily accessible … so if you make an early intervention, so to speak, you can prevent a lot of problems. And it can be very stressful, I think, for the child and parents to consult a psychologist.”* (*GP-12* ). *“Yes, (it would be helpful) to have a specialised YMHPN, because (managing adults and children) is much different … If you are keener on managing adults, like myself, then you are short-changing the children.” (MHPN-4)*

##### Facilitators described by GPs

Although GPs see the management of paediatric anxiety problems not as their primary task, GPs mentioned that they would like to have more training in paediatric anxiety problems, especially on how to recognize them. GPs would benefit from having more time and tools (e.g. patient questionnaires / patient information) to identify and help affected children. Many GPs would like to increase the availability of YMHPNs, preferably solely managing minors, in their practices. GPs had a strong wish to improve access to, and cooperation with, mental healthcare providers. They mentioned that they would like to have a better overview of the internal structure of mental healthcare institutions. Other improvements they mentioned include the reduction of waiting lists, and the enhancement of triage systems for urgent cases. In order to improve communication with mental healthcare providers, several GPs suggested creating a central point for referral and sharing patient information efficiently and confidently. GPs would benefit a great deal from possibilities to consult with an expert (e.g. child psychiatrist), especially in urgent cases. Some GPs noted that children are under considerable societal pressure, and said there was a need for a supportive, non-judgmental network of parents, friends and teachers for the affected children. Facilitators mentioned by GPs and MHPNs are shown in Table 5.

**Table 5:** Facilitators in the management of anxiety problems

GPs’ Facilitators	MHPNs’ Facilitators
**GPs’ personal and practice facilitators**	**MHPNs’ Personal and practice facilitators**
- More education - More availability of a YMHPN in practice - More time for consultations^b^ - More tools and treatment options^b^ - Availability of E-health / information websites^b^	- Continued schooling/intervision - Wish for improved recognition by GPs - More training schooling for GPs^b^ - Wish for YMHPN, dedicated only for children^a^ - More child-friendly rooms^a^
**Wishes for societal changes**	**Wishes for societal changes**
- More supportive network (e.g. parents, schools) - Less societal pressure on children	- Good cooperation with schools - Wish for improved recognition at school - Wish for less labelling as disorder by GPs and mental health care^b^ - More holistic vision in management^a^
**Facilitators external mental health care**	**Facilitators external mental health care**
- Shorter waiting lists - Improved communication with mental health care institutions - Possibility to consult mental health care expert - Mental care in close vicinity^a^ - Improved triage for referred patients^a^	- Shorter waiting lists - External mental health care easier accessible - Less bureaucracy in referral process and cooperation with external care - Wish for change in finance system of mental health care^a^

Topics mentioned by GPs and MHPNs. Main topics in bold

^a^Mentioned by one interviewee

^b^Mentioned by two interviewees

##### Facilitators described by MHPNs

Several MHPNs expressed a need for continued schooling to learn about the newest insights and to develop new tools and strategies to help affected children. Additionally, some MHPNs expressed the need for GPs to receive training in recognizing and evaluating the severity of anxiety problems. One MHPN, managing both adults and minors, suggested that practices would benefit from having a specialized YMHPN. MHPNs reported a strong wish to reduce the bureaucracy in the referral process. MHPNs would like to have a better overview of the internal structure of mental healthcare institutions. Preferably, they would like to discuss cases verbally with a designated person in the mental healthcare institution to make the referral process more efficient. The most urgent need mentioned by MHPNs is the reduction of waiting lists, especially for specialized mental health care. MHPNs said that they hoped for improvements in the recognition of anxiety problems by schools and GPs. Interestingly, some MHPNs were opposed to labelling children too quickly with an anxiety disorder diagnosis in order to avoid stigmatization, which they believed can be counterproductive.

#### What changes in the management of paediatric anxiety problems have taken place since the involvement of MHPNs and YMHPNs?

GPs reported that they remain more involved with cases of paediatric anxiety due to the introduction of MPHNs.

*“Yes, the cooperation (with the MHPN) works very well. Otherwise, you lose track of people (the referred child) after a while. Now, I keep up-to-date about what happens to them.* (*GP-6*)

GPs considered it easier to discuss cases with their MHPN than with external mental healthcare providers. Also, GPs reported remaining more involved in the treatment process after they involved a MHPN in their practice. As another positive aspect, GPs mentioned that in contrast to external care providers, MHPNs have full access to the GPs’ information system. GPs believed that care for affected children has improved since the involvement of MHPNs because affected children receive treatment in a safe and familiar environment. Also, the waiting time for the MHPNs is short, ranging from days to two weeks only. GPs thought that nowadays a substantial proportion of affected children can be treated within general practice thanks to the presence of MHPNs. GPs reported that a smaller number of referrals to external mental health services was being declined, because MHPNs were more aware of the possible referral options in their view. Additionally, MHPNs provided temporary ‘bridging’ care for children on waiting lists for external mental health care, a possibility most GPs could not offer in the past. GPs are satisfied about their cooperation with MHPNs in the management of paediatric anxiety problems. Since MHPNs could not compare the current shared management with situation before they started, they found it difficult to report on possible changes.

#### Impact of the COVID-19 pandemic

GPs and MHPNs believed that the COVID-19 pandemic did not change their management and the experienced difficulties significantly.

*“These are all things that were the case before COVID-19. And COVID-19 didn’t solve it, and I don’t expect it to become better after COVID-19.” (GP-9) “No, I believe I would have answered the questions exactly the same (if the interview had taken place before the COVID-19 pandemic).” (MHPN-3)*

Most GPs and MHPNs had the impression that paediatric anxiety problems have become more common and more severe due to the COVID-19 pandemic, which has led to delayed care and increased waiting lists. However, according to both groups of professionals, this has not led to a significant change regarding the management of affected children. GPs and MHPNs said they would not have answered our questions differently before the COVID-19 pandemic.

## Discussion

### Key findings

In this study we assessed the views of 13 GPs and 13 MHPNs on their management of anxiety problems in children and adolescents using short surveys and in-depth semi-structured interviews. As a first step, both GPs and MHPNs described exploring the problem and assessing the needs of the children and their caregivers. After exploration, GPs generally refer affected children either to MHPNs or to external mental healthcare providers. In contrast, MHPNs regularly initiate follow-up meetings with the children themselves, and they mentioned a variety of techniques and approaches which they use when confronted with paediatric anxiety problems. The barriers that GPs and MHPNs experience in the management of paediatric anxiety problems partially overlap. Important barriers, mentioned primarily by the GPs but also in part by MHPNs, include limited time and expertise in managing children (young children in particular). Important potential facilitators for a good approach in managing paediatric anxiety problems relate to the cooperation between the GP practice and external mental healthcare institutions, which needs to be improved according to both GPs and MHPNs. But importantly, the reduction of waiting lists for external mental health care for children with anxiety problems was considered most urgent by GPs as well as MHPNs.

### Strengths and limitations

Our research has several strengths. Because MHPNs became involved in managing paediatric psychosocial problems in Dutch general practice recently, only a limited number of small-scale studies assessing the role of the MHPN in managing these problems are currently available [32, 43–45]. The combination of an online survey and in-depth semi-structured interviews gave the possibility to address a wide range of topics in our research questions. Because anxiety problems include a broad spectrum of symptoms and complaints, the use of a vignette offered a well-defined starting point for our interviews. In both interviewee groups, we started analysing the interview material while still conducting the interviews. This way we were able to detect the point of data saturation, and to plan further interviews if saturation had not been reached. In both samples saturation was observed before the final interviews were conducted. The sample of participating practices included urban and more rural areas and represented patients with different socio-economic statuses. Participating GPs were diverse in terms of sex and age. Participating MHPNs showed variation regarding relevant characteristics. Only one male MHPN was interviewed, which fits with the female-dominated profession of MHPNs in the Netherlands.

Our research also has some limitations. We initially aimed to include YMHPNs. However, it became clear that only a few practices had a YMHPN who exclusively managed minors. Instead, many practices had a MHPN involved in managing both adults and minors. Although our sample reflects daily practice best, our study results may have differed if only YMHPNs solely managing minors had been included. Additionally, in the survey many GPs, as well as some MHPNs, reported having little experience with paediatric anxiety problems. However, we decided not to use GPs’ and MHPNs’ level of experience with paediatric anxiety problems as a selection criterion in order to get a full range of experiences from an unselected sample of GPs and MHPNs. Our study did not aim to address the views of affected children and parents, or the efficacy and treatment outcomes of the approach of GPs working in combination with MHPNs. Future research should, therefore, focus on addressing these issues and research questions. In our analyses we remained close to the original data and did only limited data transformation, which seemed most fitting to our aim to explore the current shared management of paediatric anxiety problems within general practice. This method is highly informative, especially in a new domain of inquiry such as our study. The chosen approach would best be considered at the rather descriptive pole in qualitative research [46]. Future research might complement our findings by more deeply exploring latent content using more interpretative analysis strategies of qualitative data.

### Comparison with existing literature

Although comparisons across countries might be limited due to significant differences between healthcare systems, some comparable initiatives to the introduction of YMHPNs in the Netherlands have taken place in other countries. In the USA, a new specialization of ‘paediatric primary care mental health specialist’ was introduced for nurse practitioners [47]. These nurse practitioners often work in a primary care practice, and are regularly involved in the management of paediatric anxiety problems. Another US initiative, a pilot study, showed that a CBT-based, nurse-led intervention was feasible in treatment of anxious children in primary care [34].

In our present research, GPs expressed missing expertise and proper tools to manage paediatric anxiety problems. This is supported by findings in our previous study: using a primary care database, we showed that GPs referred the majority of children presenting with anxiety problems, usually immediately at first consultation [48]. Also, the GPs’ prescription behaviour raised concern about their pharmacologic knowledge. GPs prescribed benzodiazepines to 1 in 11 adolescents and off-label beta-blockers to 1 in 6 adolescents in the year after presenting to their GP with an anxiety problem. SSRIs, the first-choice medication, were prescribed to only 1 in 50 adolescents [48]. Our current results are in line with other studies in which GPs felt ill-equipped to recognize and treat paediatric mental health problems, and anxiety problems in particular [38, 49, 50]. In accordance with previous literature, GPs and MHPNs mentioned that more training for GPs, especially in recognizing anxiety problems, could be a possible improvement [38, 39, 51]. In a US study, a video-based training programme improved the knowledge of paediatric residents about child anxiety disorders. In this study, participating residents improved most significantly in determining the referral urgency by recognizing ‘red flags’ [52]. This is a relevant finding since GPs have reported difficulty in determining the referral urgency, when to refer children and to whom to refer them in the case of paediatric mental health problems [40]. In our study, GPs, and to a lesser extent MHPNs, mentioned similar difficulties in the referral process. Indeed, GPs’ referral criteria for paediatric mental health problems are often much less well established in protocols or as ‘red flags’, when compared with other paediatric problems [53, 54]. In line with other research, our participants mentioned long waiting lists as the most prominent barrier for the proper treatment and management of paediatric anxiety problems [38, 39, 50].

### Implications for clinical practice and future research

The limited knowledge of GPs about paediatric anxiety problems and the difficulty they experience in recognizing these problems are reasons for concern. Future research should focus on providing effective learning and other materials for GPs. For instance, there is a lack of tools that could help improve recognition of paediatric anxiety problems in general practice, but also tools to clearly identify the need for referral, for instance by identifying relevant ‘red flags’. MHPNs reported having difficulty at times choosing the appropriate therapeutic approach. Therefore, evidence-based treatment guidelines for paediatric anxiety problems specifically aimed at the primary care settings could also help both MHPNs and GPs. However, such guidelines are lacking to date. Also, while MHPNs reported a variety of therapeutic approaches, these are not necessarily always proven to be effective. Thus, training for MHPNs should also be standardized, incorporating and promoting therapeutic approaches with proven efficacy and safety. Although GPs reported being enthusiastic about the involvement of MHPNs, it remains important to provide evidence on the effectiveness of the inclusion of MHPNs in primary care, other than the subjective evaluation of GPs. Accordingly, before advocating the introduction of MHPNs on a larger scale, it seems important to investigate whether the presence of the MHPN has indeed led to improved recognition of mental health problems, fewer referrals to external mental health care, and improved outcomes for affected children.

## Conclusion

The study demonstrates that the collaboration between GPs and MHPNs, can facilitate children’s access to treatment, with more affected children remaining treated in the general practice. Therefore, based on our interviews with GPs and MHPNs we conclude that shared efforts of GPs and MHPNs in the management of paediatric anxiety problems might help resolve at least some of the observed current problems in the management of children and adolescents with anxiety problems. To date, however, important information regarding the effectiveness of the shared efforts of GPs and MHPNs in GP practice is missing. Also, no evidence-based guidelines and trainings exist for an integrated treatment approach to paediatric anxiety problems by MHPNs and GPs at present. The findings from our interview study demonstrate an urgent need for improving the management of paediatric anxiety problems in general practice.

## Supplementary Information

**Additional file 1.**

**Additional file 2.** COREQ (COnsolidated criteria for REporting Qualitative research) Checklist.

## Data Availability

All survey findings are presented in the supplementary files. Transcripts are not available.

## References

[1] Kessler RC, Berglund P, Demler O, Jin R, Merikangas KR, Walters EE (2005). Lifetime prevalence and age-of-onset distributions of DSM-IV disorders in the National Comorbidity Survey Replication. Arch Gen Psychiatry.

[2] Polanczyk GV, Salum GA, Sugaya LS, Caye A, Rohde LA (2015). Annual research review: a meta-analysis of the worldwide prevalence of mental disorders in children and adolescents. J Child Psychol Psychiatry.

[3] Whiteford HA, Degenhardt L, Rehm J, Baxter AJ, Ferrari AJ, Erskine HE (2013). Global burden of disease attributable to mental and substance use disorders: findings from the global burden of disease study 2010. Lancet..

[4] Towe-Goodman NR, Franz L, Copeland W, Angold A, Egger H (2014). Perceived family impact of preschool anxiety disorders. J Am Acad Child Adolesc Psychiatry.

[5] Woodward LJ, Fergusson DM (2001). Life course outcomes of young people with anxiety disorders in adolescence. J Am Acad Child Adolesc Psychiatry.

[6] Copeland WE, Shanahan L, Costello EJ, Angold A (2009). Childhood and adolescent psychiatric disorders as predictors of young adult disorders. Arch Gen Psychiatry.

[7] Pape K, Bjørngaard JH, Holmen TL, Krokstad S. The welfare burden of adolescent anxiety and depression: a prospective study of 7500 young Norwegians and their families: the HUNT study. BMJ Open. 2012;2:e001942. 10.1136/bmjopen-2012-001942. 10.1136/bmjopen-2012-001942 PMC3533058 23144262

[8] James AC, Reardon T, Soler A, James G, Creswell C. Cognitive behavioural therapy for anxiety disorders in children and adolescents. Cochrane Database Syst Rev. 2020;11(11):CD013162. 10.1002/14651858.CD013162.pub2. 10.1002/14651858.CD013162.pub2 PMC8092480 33196111

[9] Wang Z, Whiteside SPH, Sim L, Farah W, Morrow AS, Alsawas M (2017). Comparative effectiveness and safety of cognitive behavioral therapy and pharmacotherapy for childhood anxiety disorders: a systematic review and meta-analysis. JAMA Pediatr.

[10] Barrett PM, Duffy AL, Dadds MR, Rapee RM (2001). Cognitive–behavioral treatment of anxiety disorders in children: long-term (6-year) follow-up. J Consult Clin Psychol.

[11] Kodal A, Fjermestad K, Bjelland I, Gjestad R, Öst L-G, Bjaastad JF (2018). Long-term effectiveness of cognitive behavioral therapy for youth with anxiety disorders. J Anxiety Disord.

[12] Saavedra LM, Silverman WK, Morgan-Lopez AA, Kurtines WM (2010). Cognitive behavioral treatment for childhood anxiety disorders: long-term effects on anxiety and secondary disorders in young adulthood. J Child Psychol Psychiatry.

[13] Chavira DA, Stein MB, Bailey K, Stein MT (2004). Child anxiety in primary care: prevalent but untreated. Depress Anxiety.

[14] Emslie GJ (2008). Pediatric anxiety — underrecognized and undertreated. N Engl J Med.

[15] Richardson LP, Russo JE, Lozano P, McCauley E, Katon W (2010). Factors associated with detection and receipt of treatment for youth with depression and anxiety disorders. Acad Pediatr.

[16] Reardon T, Harvey K, Baranowska M, O’Brien D, Smith L, Creswell C (2017). What do parents perceive are the barriers and facilitators to accessing psychological treatment for mental health problems in children and adolescents? A systematic review of qualitative and quantitative studies. Eur Child Adolesc Psychiatry..

[17] Chavira DA, Bantados B, Rapp A, Firpo-Perretti YM, Escovar E, Dixon L (2017). Parent-reported stigma and child anxiety: a mixed methods research study. Child Youth Serv Rev.

[18] Zachrisson HD, Rödje K, Mykletun A (2006). Utilization of health services in relation to mental health problems in adolescents: a population based survey. BMC Public Health.

[19] Radez J, Reardon T, Creswell C, Lawrence PJ, Evdoka-Burton G, Waite P (2021). Why do children and adolescents (not) seek and access professional help for their mental health problems? A systematic review of quantitative and qualitative studies. Eur Child Adolesc Psychiatry.

[20] Aydin S, Crone MR, Siebelink BM, Vermeiren RRJM, Numans ME, Westenberg PM (2020). Recognition of anxiety disorders in children: a cross-sectional vignette-based survey among general practitioners. BMJ Open.

[21] Wren FJ, Scholle SH, Heo J, Comer DM (2003). Pediatric mood and anxiety syndromes in primary care: who gets identified?. Int J Psychiatry Med.

[22] Neil L, Smith M (2017). Teachers’ recognition of anxiety and somatic symptoms in their pupils. Psychol Sch.

[23] Remschmidt H, Belfer M (2005). Mental health care for children and adolescents worldwide: a review. World Psychiatry.

[24] Belfer ML (2008). Child and adolescent mental disorders: the magnitude of the problem across the globe. J Child Psychol Psychiatry.

[25] Das JK, Salam RA, Lassi ZS, Khan MN, Mahmood W, Patel V (2016). Interventions for adolescent mental health: an overview of systematic reviews. J Adolesc Health.

[26] CAMHS. CYP IAPT principles in CAMH services values and standards: ‘delivering with and delivering well’. CAMHS Press. 2014.

[27] Tyler ET, Hulkower RL, Kaminski JW. Behavioral health integration in pediatric primary care. Milbank Memorial Fund. 2017;15:1–24

[28] Rickwood D, Paraskakis M, Quin D, Hobbs N, Ryall V, Trethowan J (2019). Australia's innovation in youth mental health care: the headspace Centre model. Early Interv Psychiatry..

[29] Bassilios B, Nicholas A, Reifels L, King K, Spittal MJ, Fletcher J (2016). Improving access to primary mental health care for Australian children. Aust N Z J Psychiatry.

[30] Sarvet B, Gold J, Bostic JQ, Masek BJ, Prince JB, Jeffers-Terry M (2010). Improving access to mental health care for children: the Massachusetts child psychiatry access project. Pediatrics..

[31] Magnée T, de Beurs DP, Schellevis FG, Verhaak PF (2019). Ontwikkelingen in de Nederlandse huisartsenzorg voor psychische problemen: een overzicht van recente studies. Tijdschr Psychiatr.

[32] Zwaanswijk M, Geuijen P, Boelhouwer M, Spijk-de Jonge M, Serra M (2020). Verwijspatronen bij praktijkondersteuners jeugd. Huisarts Wet.

[33] Chavira DA, Drahota A, Garland AF, Roesch S, Garcia M, Stein MB (2014). Feasibility of two modes of treatment delivery for child anxiety in primary care. Behav Res Ther.

[34] Kozlowski JL, Lusk P, Melnyk BM (2015). Pediatric nurse practitioner management of child anxiety in a rural primary care clinic with the evidence-based COPE program. J Pediatr Health Care.

[35] Weersing VR, Brent DA, Rozenman MS, Gonzalez A, Jeffreys M, Dickerson JF (2017). Brief behavioral therapy for pediatric anxiety and depression in primary care: a randomized clinical trial. JAMA Psychiatry.

[36] Guest G, Bunce A, Johnson L (2006). How many interviews are enough? An experiment with data saturation and variability. Field Methods.

[37] Vasileiou K, Barnett J, Thorpe S, Young T (2018). Characterising and justifying sample size sufficiency in interview-based studies: systematic analysis of qualitative health research over a 15-year period. BMC Med Res Methodol.

[38] O'Brien D, Harvey K, Creswell C. Barriers to and facilitators of the identification, management and referral of childhood anxiety disorders in primary care: a survey of general practitioners in England. BMJ Open 2019;9(4):e023876-e. 10.1136/bmjopen-2018-023876 PMC6501977 31015266

[39] Leahy D, Schaffalitzky E, Saunders J, Armstrong C, Meagher D, Ryan P (2018). Role of the general practitioner in providing early intervention for youth mental health: a mixed methods investigation. Early Interv Psychiatry.

[40] Hinrichs S, Owens M, Dunn V, Goodyer I. General practitioner experience and perception of child and adolescent mental health services (CAMHS) care pathways: a multimethod research study. BMJ Open 2012;2:e001573. 10.1136/bmjopen-2012-001573. 10.1136/bmjopen-2012-001573 PMC3533003 23148343

[41] Mayring P, Fenzl T, editors. Qualitative content analysis program qcamap–an open access text analysis software. 15th Biennial EARLI Conference for Research on Learning and Instruction, Munich, Germany; 2016.

[42] Boeije H. Analysis in qualitative research. London: Sage publications; 2009.

[43] Jonker T, Remmelts H, Huyghen A-M, van der Woude G, Knot-Dickscheit J (2020). Ervaringen en perspectief van de POH-Jeugd. Huisarts Wet..

[44] Jonker TJ, Knot-Dickscheit J, Huyghen A. De praktijkondersteuner huisarts-jeugd: een verkennende studie. Groningen: University of Groningen; 2017.

[45] Jonker TJ, Knot-Dickscheit J, Huyghen A (2017). De praktijkondersteuner huisarts-jeugd: een verkennende studie.

[46] Kiger ME, Varpio L (2020). Thematic analysis of qualitative data: AMEE guide no. 131. Medical teacher.

[47] Hawkins-Walsh E, Van Cleve SN (2019). A job task analysis of the expanding role of the pediatric mental health specialist and the nurse practitioner in pediatric mental health. J Pediatr Health Care.

[48] Koet LBM, de Schepper EIT, Bohnen AM, Bindels PJE, Gerger H. Anxiety problems in children: a population-based cohort study on incidence and management. Br J Gen Pract. 2022;72(719):e405–e412. 10.3399/BJGP.2021.0557. 10.3399/BJGP.2021.0557 PMC9037188 35440466

[49] Zarafshan H, Wissow LS, Shahrivar Z, Mojtabai R, Khademi M, JafariNia M (2021). Children and adolescents' mental health in Iran's primary care: perspectives of general practitioners, school staff and help seekers. Glob Soc Welf.

[50] O'Brien D, Harvey K, Howse J, Reardon T, Creswell C (2016). Barriers to managing child and adolescent mental health problems: a systematic review of primary care practitioners' perceptions. Br J Gen Pract.

[51] Horwitz SM, Storfer-Isser A, Kerker BD, Szilagyi M, Garner A, O'Connor KG (2015). Barriers to the identification and management of psychosocial problems: changes from 2004 to 2013. Acad Pediatr.

[52] Nicholas N, Pincus D, Perrin E, Bair-Merritt M (2020). Identifying and making recommendations for pediatric anxiety disorders in primary care settings: a video-based training. MedEdPORTAL..

[53] Ramanayake RPJC, Basnayake BMTK (2018). Evaluation of red flags minimizes missing serious diseases in primary care. J Family Med Prim Care.

[54] AACAP. Recommendations for pediatricians, family practitioners, psychiatrists, and non-physician mental health practitioners. J Am Acad Child Adolesc Psychiatry. 2022. [Cited 2022 Jul 7]. Available from: https://www.aacap.org/aacap/Member_Resources/Practice_Information/When_to_Seek_Referral_or_Consultation_with_a_CAP.aspx.

