# Supplementary material for: GPs’ and practice nurses’ views on their management of paediatric anxiety problems: an interview study
Source: BMC Prim Care. 2022 Sep 12;23:235. doi: 10.1186/s12875-022-01802-y (PMC9465156; doi:10.1186/s12875-022-01802-y)
Supplement: Supplementary file 1 — Additional file 1. [file 12875_2022_1802_MOESM1_ESM.docx]

**Supplementary file S1a GPs’ survey**

| Participant number | **Age**  (years) /  **Sex**  (M/F) | **Years working as GP** | **Full time** | **Number of children in practice** | **Experience with diagnosing anxiety symptoms in children** | **Experience treating anxiety problems** **in children** | **Average socio-economic status (SES) of patient population** | **Since when can you refer children to MHPN?** | **Can you refer to a neighbour-hood team?** | **If yes, since when?** | **Approach in children with anxiety problems** | **To whom did you usually refer?** |
| --- | --- | --- | --- | --- | --- | --- | --- | --- | --- | --- | --- | --- |
| **1** | 38  M | 10 | Yes | Average | Limited | Very limited | Average | 2020 | Yes | 2018 | Direct referral for additional examination and treatment | Specialised mental health care |
| **2** | 56  M | 30 | Yes | Average | Neutral | Neutral | Average | 2014 | No | - | Investigate the problem yourself after which referral for treatment | MHPN |
| **3** | 46  M | 20 | Yes | Low | Limited | Limited | Low | 2018 | Yes | 2000 | Investigate the problem yourself after which referral for treatment | MHPN |
| **4** | 49  M | 19 | Yes | Average | Neutral | Neutral | Average | 2015 | Yes | 2018 | Investigate the problem yourself after which referral for treatment | MHPN |
| **5** | 32  M | 2.5 | No | Average | Neutral | Neutral | Average | 2016 | No | - | *Others: Often in consultation with MHPN to determine management* | MHPN |
| **6** | 48  M | 15 | Yes | Average | Neutral | Neutral | Average | 2017 | No | - | Direct referral for additional examination and treatment | Child psychologist |
| **7** | 51  F | 20 | No | Average | Neutral | Neutral | Average | 2018 | Yes | 2015 | *Others: all of the options* | MHPN |
| **8** | 50  F | 15 | Yes | Average | Limited | Limited | High | 2019 | Yes | 2011 | Investigate the problem yourself after which referral for treatment | MHPN |
| **9*** | 51  M | 22 | Yes | Low | Limited | Very limited | Average | - | Yes | 2018 | Wait and see | Child psychologist |
| **10** | 41  M | 11 | No | Average | A lot | Neutral | Low | 2020 | Yes | 2017 | Investigate the problem yourself after which referral for treatment | Child psychologist |
| **11** | 52  F | 20 | No | Low | Very limited | Very limited | Average | 2020 | Yes | 2016 | Direct referral for additional examination and treatment | Specialised mental health care |
| **12** | 48  F | 18 | No | High | A lot | Neutral | Average | 2019 | No | - | Investigate the problem yourself after which referral for treatment | Child psychologist |
| **13** | 44  F | 14 | No | Average | Neutral | Neutral | Average | 2020 | Yes | 2018 | *Others: MHPN* | MHPN |

**GP 9 could not refer to a MHPN that managed minors*

**Supplementary file S1b MHPNs’ survey**

| **Participant number** | **Age**  (years) /  **Sex**  (M/F) | **Education** | **Exclusively treat**s **children and adolescents?** | **Years working as a MHPN** | **Experience diagnosing anxiety** problems **in children** | **Experience treating anxiety** problems **in children** | **Average socio-economic status (SES) of patient population** | **Can you refer to a neighbor-hood team?** | **If** yes**, since when?** | **Approach in children with anxiety problems** | **To whom did you usually refer?** |
| --- | --- | --- | --- | --- | --- | --- | --- | --- | --- | --- | --- |
| **1** | 36  F | UAS* Social work | Yes | 1 | Limited | Very much | Average | Yes | 2021 | Investigate the problem myself  and start treatment | Specialized Mental Health Services |
| **2** | 48  F | UAS* master | Yes | 1 | Neutral | Limited | Average | Yes | 2021 | Investigate the problem myself  and referral for treatment | Specialized Mental Health Services |
| **3** | 34  F | UAS* Social work / MHPN | No | 2 | Limited | Neutral | Low | Yes | 2019 | Investigate the problem myself  and start treatment | Specialized Mental Health Services |
| **4** | 52  F | UAS* | No | 4 | Limited | Very limited | Average | No | na | Investigate the problem myself  and referral for treatment | Child psychologist |
| **5** | 54  F | Post-graduate MHPN | No | 1,5 | Limited | Limited | Average | Yes | 2015 | Investigate the problem myself  and referral for treatment | Other: non-specialized Mental Health Care |
| **6** | 58  F | UAS* | No | 10 | Neutral | Neutral | Average | Yes | 2016 | Investigate the problem myself  and referral for treatment | Child psychologist |
| **7** | 52  F | UAS* social pedagogy / grief therapy / trauma informed therapy | Yes | 3 | Neutral | Much | Average | Yes | 2018 | Other: All of the four answers | Other*** |
| **8** | 34  F | UAS* nursing / post-graduate MPHN | No | 3 | Limited | Limited | Average | Yes | 2018 | Investigate the problem myself  and referral for treatment | Child psychologist |
| **9** | 30  F | University psychology | No | 3 | Neutral | Neutral | Average | Yes | 2020 | Other: investigate more and refer or treat depending on severity and reason of consultation | Child psychologist |
| **10** | 60  F | UAS* social work | No | 8 | Much | Much | Average | Yes | 2019 | Investigate the problem myself  and start treatment | Child psychologist |
| **11** | 48  F | UAS* | No | 2 | Very limited | Very limited | Low | Yes | 2019 | Direct referral for additional  examination and treatment | Neighborhood team |
| **12** | 56  M | UAS* social psychiatric nursing / MPHN | No | 7 | Limited | Much | Average | Yes | 2018 | Investigate the problem myself  and referral for treatment | Child psychologist |
| **13** | 50 | University psychology | Yes | 2 | Neutral | Much | Average | No | na | Other: treat child depending on reasons of the anxiety | Child psychologist |

**University of applied sciences **none of participants reported working ≥36 hours/week as MHPN ***”Depending on the nature of the complaints! Sometimes I treat patients myself in general practice (trauma- informed working), sometimes in collaboration with physiotherapy. Very often I provide ‘bridging’ care to children on waiting list.”*

**Supplementary file S2 Main topics and subtopics per research question**

***Supplementary file S2a*** *Code tree: What is the current management of paediatric anxiety problems by GPs? (research question: orange, main topic: blue, topic: grey, subtopic: white. * Mentioned by one interviewee ** mentioned by two interviewees)*

***Supplementary file S2b*** *Code tree: What are barriers in the management of anxiety problems in children experienced by GPs? (research question: orange, main topic: blue, topic: grey, subtopic: white. * Mentioned by one interviewee ** mentioned by two interviewees)****Supplementary file S2c*** *Code tree: What are facilitators in the management of paediatric anxiety problems experienced by GPs? (research question: orange, main topic: blue, topic: grey, * Mentioned by one interviewee ** mentioned by two interviewees)*

***Supplementary file S2d*** *Code tree: According to GPs,* What changes in the management of paediatric anxiety problems have taken place since the involvement of MHPNs? *(research question: orange, main topic: blue, topic: grey, subtopic: white. * Mentioned by one interviewee ** mentioned by two interviewees)*

***Supplementary file S2e*** *Code tree: other identified topics in the interviews with GPs (main topic: blue, topic: grey, subtopic: white. * Mentioned by one interviewee ** mentioned by two interviewees)*

***Supplementary file S2f*** *Code tree: What is the current management of paediatric anxiety problems by MHPNs? (research question: orange, main topic: blue, topic: cream, sub-topic: white. * Mentioned by one interviewee ** mentioned by two interviewees)*

***Supplementary file S2g*** *Code tree: What are barriers in the management of pediatric anxiety problems experienced by MHPNs (research question: orange, main topic: blue, topic: cream, sub-topic: white. * Mentioned by one interviewee ** mentioned by two interviewees)*

***Supplementary file S2h*** *Code tree: facilitators in management of paediatric anxiety problems experienced by MHPNs (research question: orange, main topic: blue, topic: cream, sub-topic: white. * Mentioned by one interviewee ** mentioned by two interviewees)*

***Supplementary file S2i*** *Code tree: According to MHPNs, what changes in the management of paediatric anxiety problems have taken place since their involvement? (research question: orange, main topic: blue, topic: cream, sub-topic: white. * Mentioned by one interviewee ** mentioned by two interviewees)*

***Supplementary file S2j*** *Code tree: Other identified topics in interviews with MHPNs (research question: orange, main topic: blue, topic: cream, sub-topic: white. * Mentioned by one interviewee ** mentioned by two interviewees)*

**Supplementary file S4: *Survey: Questionnaire for GPs / MHPNs***

|  | Participant characteristics: | | |
| --- | --- | --- | --- |
| What is your age? | |  | **free text** |
| What is your sex? | |  | M/F/other |
| How many years are you working as GP? *¥* | |  | **free text** |
| How many years are you working as MHPN? *±* | |  | **free text** |
| Type of education / highest grade obtained? *±* | |  | **free text** |
| In your position as (youth) mental health practice nurse,  Do you solely manage children and adolescents? *±* | |  | Yes / No, also adults |
| Do you currently work full time as MHPN/GP  (≥36hours/week)? | |  | Yes / No |
|  | **Practice characteristics:** | | |
| What is the average social economic status of your patient population? | |  | Relative low / Average / Relative high |
| How is your patient population characterized? | |  | Relatively few children/ average number of children/ relatively many children |
| Since when can you refer a child with psychosocial problems to a (youth) mental health practice nurse? *¥* | |  | **free text** |
| Do you have the possibility in your practice to refer children with psychosocial problems to the ‘neighbourhood team’ | |  | Yes / No |
| If yes, since what year can you refer to the ‘neighbourhood team’ | |  | **free text** |
|  | **Child population and approach:** | | |
| Do you have experience diagnosing anxiety problems in children? | |  | Very limited / limited / neutral / much/ very much |
| Do you have experience treating anxiety problems in children? | |  | Very limited / limited / neutral / much / very much |
| What did you usually do when confronted with a child with excessive anxiety problems? | |  | Direct referral for additional diagnostics and treatment/ Explore the case oneself, and subsequently refer for treatment/ Explore the case oneself, and subsequently initiate treatment oneself/ Wait and see/ Other, namely.. **free text** |
| In you referred a child with anxiety problems, to whom did you usually refer? | |  | (Y)MHPN*¥ /* the ‘neighourhood team’ / Child psychologist / Specialised mental health care – other, namely **free text** |

*¥ only in GPs questionnaire*

*± only in MHPNs questionnaire*

**Supplementary file S5a: *Vignette: Case description of child with anxiety problems***

English translation:

Eva is a 13-year-old girl, who visits your practice together with her mother. They want to discuss with you some of her problems that have increased over the past year. The mother tells you that Eva has always been a shy girl fearing rejection. At her previous school, she had several friends who she got on well with. At her new school, however, her mother tells, Eva is unable to connect with the children in her class. Her mother asks Eva to tell about a recent incident in school. Eva tells you she had to give a presentation last week. During the presentation, she burst out into tears, without knowing why. After a conversation with her teacher, her mother found out that Eva had not yet made new friends in her new school and that she often spends breaks alone. According to her mother, Eva does not respond to birthday invitations. She recently took a trial lesson at the hockey club, but did not continue playing hockey. Eva tells you she believes her classmates don't like her. According to her mother, Eva has been complaining occasionally about stomach ache when she is at home lately. Usually, she spends her evenings alone in her room with her cat. Her mother attributed this to her introverted personality. Eva gets good grades at school. But after the latest outburst, her teacher informed Eva's mother about her situation. They are now consulting you, and her mother asks you for advice.

**Supplementary file S5b: Guide for interview questions with GPs** (translation)

*How do you usually treat a child with problems similar to those described in the vignette in*

*general practice?*

- *Why?*
- *In this vignette, what would have to change to opt for a referral?*

*What do you find most helpful when you are confronted with such a case in coming to a decision*

*how to treat such a child or what exactly to recommend/advise?*

- *Why?*
- *More education/more time/skills/a MHPN/sufficient space at the MHPN for long-term*

*treatment?*

*What are difficulties which you experience when confronted with such a case?*

*How would you define your role as GP/MHPN in the treatment / management of such a case?*

- *Do you see the MHPN / yourself as part of general practice or rather as an external*

*health care service?*

- *Do you think the treatment / management of anxiety problems as described in the case vignette in children and adolescents in general practice has changed since the involvement of the MHPN?*
- *What has changed?*
- *Why do you think this occurred?*
- *Is this better or worse than before?*

*If you could make a wish, what would be required for improving treatment of paediatric anxiety in*

*general practice in your view?*

- *Why?*
- *What is required to improve the quality of collaborations with other mental health care*

*services?*

*What has changed in the management of anxiety problems in your practice / your experience*

*during the last year of the pandemic?*

- *Do you have the impression you saw more or less children and adolescents with anxiety-related problems as the one described in the vignette during the last year?*
- *Do you think these children / adolescents got adequate attention / treatment during the last year of pandemic?*

*Do you think you would have answered the questions differently if you were asked before the*

*COVID pandemic?*

- *What would be different?*

**Supplementary file S5c: Guide for interview questions with MHPNs** (translation)

*How do you usually treat a child with problems similar to those described in the vignette in general practice?*

- *Why?*
- *In this vignette, what would have to change to opt for a referral?*

*What do you find most helpful when you are confronted with such a case in coming to a decision how to treat such a child or what exactly to recommend/advise?*

- *Why?*
- *More education/more time/do you feel competent?*

*What are difficulties which you experience when confronted with such a case?*

- *Why?*
- *Do you feel competent to treat anxiety problems in children and adolescents yourself?*
- *What is a sign for you to decide to refer to secondary care/a mental health specialist?*

*How would you define your role as MHPN in the treatment / management of such a case?*

- *Do you see yourself, as a MHPN, as part of general practice or rather as an external health care service?*
- *What do you think about how the patient sees you?*

*What have you changed in the treatment of paediatric anxiety problems as described in the vignette in general practice since your involvement as MHPN?*

- *Why did you change this?*
- *Do you reckon this to be an improvement?*

*What if you could make a wish, would be required for improving treatment of paediatric anxiety problems in general practice in your view?*

- *Why?*

*What is required to improve the quality of collaborations with other mental health care services?*

- *What does the collaboration with the ‘neighbourhood team’ look like?*

*What has changed in the management of paediatric anxiety problems in your practice / your experience during the last year of the pandemic?*

- *Do you have the impression you managed more or less children and adolescents with anxiety-related problems as the one described in the vignette during the last year*
- *Do you think these children / adolescents got adequate attention / treatment during the last year of pandemic?*

*Do you think you would have answered the questions differently if you were asked before the COVID pandemic?*

- *What would be different?*
